# Selected Biomarkers Correlate with the Origin and Severity of Sepsis

**DOI:** 10.1155/2018/7028267

**Published:** 2018-03-27

**Authors:** Michal Holub, Olga Džupová, Michaela Růžková, Alžběta Stráníková, Eva Bartáková, Jan Máca, Jiří Beneš, Heiko Herwald, Ondřej Beran

**Affiliations:** ^1^Department of Infectious Diseases, First Faculty of Medicine, Charles University and Military University Hospital Prague, U Vojenské nemocnice 1200, 169 02 Praha 6, Czech Republic; ^2^Department of Infectious Diseases, Third Faculty of Medicine, Charles University and Na Bulovce Hospital, Budínova 2, 180 81 Praha 8, Czech Republic; ^3^Department of Anesthesiology and Intensive Care Medicine, University Hospital of Ostrava, 17. listopadu, 708 52 Ostrava, Czech Republic; ^4^Department of Clinical Sciences, Division of Infection Medicine, Lund University, Sölvegatan 19, 221 00 Lund, Sweden

## Abstract

The microbial etiology and source of sepsis influence the inflammatory response. Therefore, the plasma levels of cytokines (IL-6, IL-8, and IL-10), chemokines (CCL2/MCP-1, MIP-1*β*), heparin-binding protein (HBP), soluble CD14 (sCD14), and cortisol were analyzed in blood from septic patients obtained during the first 96 hours of intensive care unit hospitalization. The etiology was established in 56 out of a total of 62 patients enrolled in the study. Plasma concentrations of MCP-1, sCD14, IL-6, and IL-10 were significantly higher in patients with community-acquired pneumonia (CAP; *n* = 10) and infective endocarditis (IE; *n* = 11) compared to those with bacterial meningitis (BM; *n* = 18). Next, cortisol levels were higher in IE patients than in those with BM and CAP, and at one time point, cortisol was also higher in patients with gram-negative sepsis when compared to those with gram-positive infections. Furthermore, cortisol and MCP-1 levels correlated positively with the daily measured SOFA score. In addition, HBP levels were significantly higher in patients with IE than in those with BM. Our findings suggest that MCP-1, sCD14, IL-6, IL-10, cortisol, and HBP are modulated by the source of sepsis and that elevated MCP-1 and cortisol plasma levels are associated with sepsis-induced organ dysfunction.

## 1. Introduction

Sepsis represents the main cause of death from infectious diseases. Current mainstays of sepsis therapy are early recognition and timely antibiotic administration [1]. Since the results of microbiology tests are usually significantly delayed and since they are negative in almost 40% of patients, the selection of the antibiotic therapy is usually empirical, based on the presumed source of the sepsis and the epidemiological situation (i.e., community- versus hospital-acquired infection) [2, 3]. This often results in the use of combinations of broad-spectrum antibiotics, which can lead to colonization by multiresistant flora and other side effects, including *Clostridium difficile* infection [4, 5]. Therefore, the prevention of the unnecessary use of broad-spectrum antibiotics and the promotion of the use of narrow-spectrum antibiotics are of paramount importance.

Blood culture remains the standard test for sepsis, although the results are not usually available until 24 to 48 hours after the collection. Thus, blood biomarkers may sometimes provide faster information for the assessment of the microbial etiology of the infection, thereby helping select the appropriate empiric antimicrobial therapy. A good example of a reliable biomarker is procalcitonin (PCT), which is intensively upregulated in gram-negative sepsis and community-acquired pneumonia (CAP) caused by typical pathogens, mainly *Streptococcus pneumoniae* [6, 7]. Similarly, interleukin- (IL-) 6 in blood has been found in 10-fold higher concentrations in neonates with gram-negative sepsis than in children with gram-positive sepsis, and in adults with CAP due to typical bacterial pathogens in comparison to those with atypical or viral pneumonia [8, 9]. Although these data seem to be promising, the high concentrations of circulating biomarkers observed in septic patients were evaluated mostly for prognostic reasons, and only a limited number of studies analyzed their value for the etiologic diagnosis of sepsis [10, 11]. Furthermore, there is a lack of data about the influence of the source of sepsis on blood biomarkers that might be helpful in source control and in the selection of an appropriate antimicrobial therapy [12].

Therefore, we analyzed selected inflammatory parameters obtained from intensive care unit (ICU) patients with sepsis during the first four days (96 hours) of hospitalization and assessed their associations with the bacterial etiology and source of sepsis.

## 2. Patients and Methods

The study was approved by the local ethics committee (IRB00002721) and performed in accordance with the Convention on Human Rights and Biomedicine (Oviedo 1997). The study subjects were enrolled after signing the written informed consent. Patients not able to sign the consent were enrolled by two attending physicians; however, their specimens were analyzed only if they signed the consent after their recovery. A prospective cohort study was carried out between November 2010 and November 2014 in the ICU of the Department of Infectious, Tropical and Parasitic Diseases, Na Bulovce Hospital, Prague, Czech Republic.

The inclusion criteria were the standard diagnostic criteria for sepsis valid at the time of the beginning of the study [13]. The exclusion criteria consisted of severe immunodeficiency, including HIV infection, oncologic disease, transfer from another hospital, a life expectancy < 48 hours, and a duration of antimicrobial therapy > 24 hours prior to enrollment into the study. The severity of illness was assessed by the Acute Physiology and Chronic Health Evaluation (APACHE) II score using the worst clinical and laboratory values during the first 24 hours after admission, and the Sequential Organ Failure Assessment (SOFA) score was calculated daily for four consecutive days to evaluate organ dysfunction. The etiologic diagnosis was made by the detection of pathogenic bacteria in blood cultures, cerebrospinal fluid (CSF), or urine; by the detection of bacterial or viral DNA in clinical specimens; or by the positivity for specific acute antibodies in the case of Legionnaire's disease or influenza. The sources of sepsis were identified by CSF analysis, chest X-ray, esophageal echocardiography, abdominal ultrasonography, or CT scan of the chest and/or abdomen. Altogether, 62 septic patients were enrolled into the study. Their demographic and clinical data are presented in Table 1. For the specialized laboratory tests, the venous blood was collected into S-Monovette tubes with heparin (Sarstedt AG & Co., Nümbrecht, Germany). All blood samples were immediately centrifuged and stored at −80°C until further analysis.

Cytokines and chemokines, namely, IL-6, IL-8, IL-10, macrophage inflammatory protein- (MIP-) 1*β*, and monocyte chemoattractant protein- (MCP-) 1, were analyzed using FlowCytomix™ multiplex analysis (eBioscience, Bender MedSystems GmbH, Vienna, Austria) and six-color flow cytometry (BD FACSCanto™ II, BD Biosciences, CA, San Jose, USA) with FACSDiva™ software (BD). Next, commercial enzyme-linked immunosorbent assays (ELISA) were used to measure serum levels of soluble CD14 (R&D Systems, Minneapolis, MN, USA), and an in-house ELISA was used for the heparin-binding protein (HBP) analysis; the method for HBP detection is described elsewhere [14]. The analysis of cortisol was performed on an Architect i2000™ immunochemistry analyzer (Abbott, Chicago, IL, USA) using the fluorescence polarization immunoassay FPIA (Abbott).

Statistical analyses were performed using STATISTICA 12 Stat Soft. The data are presented as medians (quartiles or interquartile range). Levels that could not be detected were assigned values equal to the lower detection limit of the test. Differences between analyzed parameters in the groups were tested using the Mann–Whitney *U* test, and for comparisons between values within the etiology, *α* < 0.05. Further, the Kruskal-Wallis test was used for multiple comparisons at the *α* < 0.05 level. Spearman's correlation test was used for the determination of correlations between the variables. A certified biostatistician (Dipl.-Ing. Nikol Kaspříková) confirmed the use of the statistical methods.

## 3. Results

### 3.1. Patients

**Figure 1:**
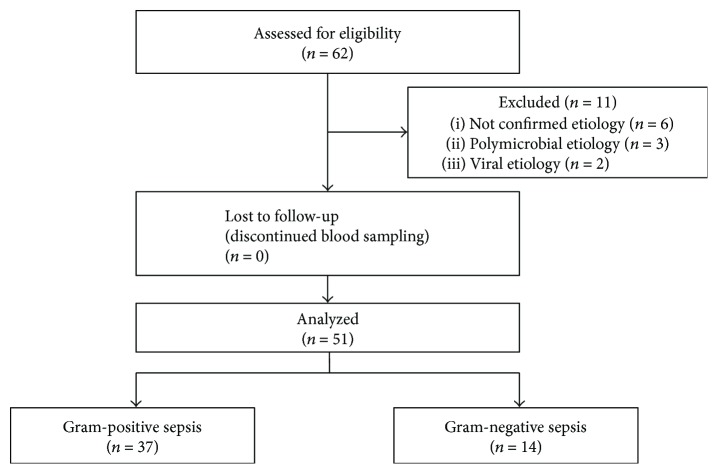
Flow chart of the patient selection process for the analysis based on confirmed gram-positive and gram-negative etiology of sepsis.

Out of the 62 enrolled subjects, 56 patients had a confirmed microbial etiology of sepsis, and 51 of them had confirmed either gram-positive or gram-negative etiology of sepsis.  Figure 1 demonstrates the process of their selection. We analyzed data from 37 patients suffering from sepsis caused by gram-positive pathogens. The most frequent clinical diagnosis of the patients with gram-positive sepsis was bacterial meningitis (BM; *n* = 12), followed by infective endocarditis (IE; *n* = 11), CAP (*n* = 5), severe soft tissue infection (SSTI; *n* = 3), vertebral osteomyelitis (*n* = 2), biliary sepsis (*n* = 2), acute bacterial epiglottitis (*n* = 1), and sepsis of unknown source (*n* = 1). Additional 14 patients were diagnosed with sepsis due to gram-negative pathogens. The clinical diagnoses of these patients were urosepsis (*n* = 6) followed by biliary sepsis (*n* = 2), BM (*n* = 2), CAP (*n* = 2), meningococcal sepsis (*n* = 1), and acute enteritis (*n* = 1). The comparison of the baseline characteristics of the patients diagnosed with gram-positive and gram-negative sepsis is shown in Table 2. Next, for the evaluation of the influence of the source of sepsis, the data of patients diagnosed with BM were compared to the data of patients with IE and those with CAP. Baseline and etiological characteristics of these patient groups are presented in Tables 3 and 4. Initial total and system-related SOFA score values in the groups of patients evaluated based on source and etiology of sepsis are presented in Table 5.

### 3.2. Detection of Inflammatory Mediators in the Specimens

Altogether, we collected 1948 specimens, and the selected analytes were detected in 1687 (86.6%) samples. The plasma levels of MCP-1, sCD14, and HBP were detectable in all specimens collected during the study period. The plasma levels of cortisol and MIP-1*β* were detectable in most specimens, followed by the levels of IL-8, IL-6, and IL-10. The exact data are presented in Table 6.

### 3.3. Inflammatory Parameters in Gram-Positive and Gram-Negative Sepsis

Comparison of the biomarkers between patients with gram-positive and gram-negative sepsis demonstrated a very limited number of significant differences. Notably, plasma levels of cortisol were significantly higher in patients with gram-negative sepsis than in those with gram-positive sepsis only on day 2 of the study. Although the MCP-1 concentration demonstrated a similar trend in patients with gram-negative infections over the whole study period, it did not reach statistical significance. The median concentrations of the examined biomarkers and their statistical comparisons are presented in Table 7.

### 3.4. Inflammatory Parameters and the Source of Sepsis

The plasma levels of MCP-1, sCD14, cortisol, IL-6, IL-10, and HBP were significantly decreased in patients with BM in comparison to those with either IE or CAP. A significant difference between patients with IE and CAP was found in cortisol plasma levels only during one time point, namely, on day 4. Notably, patients with BM in comparison to those with CAP and IE had significantly decreased plasma levels of MCP-1 at all time points and sCD14 on days 2, 3, and 4. The level of sCD14 on day 1 was lower in patients with BM compared to those with CAP. The plasma cortisol levels were lower in patients with BM in comparison to those with IE on days 2, 3, and 4 and on day 3 in comparison to those with CAP. Similar findings in patients with BM were also demonstrated for IL-10 in comparison to those with CAP on days 1, 3, and 4 and in comparison to those with IE on days 1 and 2. Patients with BM also had significantly lower IL-6 plasma levels in comparison to those of patients with CAP on days 2 and 3. Additionally, the level of HBP was significantly lower in patients with BM when compared to those with IE on days 1 and 3. The biomarker concentrations in the evaluated sources of sepsis and their statistical comparisons are presented in Table 8.

### 3.5. Correlations among the Laboratory Parameters and the SOFA Score

We assessed the correlation among the SOFA score and the inflammatory parameters with a detectability above 90%. Two of the selected sepsis biomarkers—MCP-1 and cortisol—demonstrated consistently significant correlations with the daily SOFA score over the study period. These data are presented in Table 9.

## 4. Discussion

In the current study, we observed significant relationships between plasma levels of MCP-1, sCD14, HBP, and cortisol and the source of sepsis, whereas the well-established inflammatory cytokines (i.e., IL-6 and IL-10) demonstrated relatively low detectability in our cohort of septic patients. Furthermore, plasma MCP-1 and cortisol levels were significantly correlated with the SOFA score, and cortisol was also correlated with the gram-negative etiology of sepsis.

Blood concentrations of chemokines had already been evaluated with a cytometric bead array in a study that enrolled 89 children with acute bacterial infections, which included patients with CAP, sepsis, and bacterial abscesses [15]. In that study, the median plasma MCP-1 levels in all three cohorts were significantly lower than those observed in 20 healthy controls (i.e., 24.9 pg/mL), which authors explained by a relatively long duration of the illness, with a median of six days before enrollment in the study. In any case, these concentrations were low. In another study, an analysis of baseline cytokine concentrations with a Luminex™ assay demonstrated serum MCP-1 levels of 62.8 pg/mL in healthy males and 55.4 pg/mL in healthy females [16]. Regarding IE and CAP, our findings are in agreement with those of a study performed in 137 patients with CAP that demonstrated mean plasma MCP-1 levels of 803.2 pg/mL after admission [17]. The variability of the concentrations found in different studies can be explained by the specific cohorts of patients and the use of different analytic methods, that is, Luminex, ELISA, or cytometric arrays. Additionally, a blood collection method can be responsible for the differences among detected concentrations, because in some studies plasma was used and in others serum was used. On the other hand, in our previous study in which serum concentrations of MCP-1 were detected with the Luminex method in 21 adult patients with bacterial infection, almost the same concentrations were found compared to the current study using plasma and the cytometric assay method [18]. This may suggest that the source of sepsis is a more important factor than the analytic method used for MCP-1 detection. In addition, the positive correlation of the plasma MCP-1 levels with the SOFA score in all 62 enrolled patients may indicate the importance of this chemokine in sepsis outcome. This has already been described in children with meningococcal sepsis whose serum MCP-1 levels correlated positively (*r* = 0.68) with their SOFA score and in adults who died of sepsis and who had significantly higher serum MCP-1 levels than the levels in survivors [19, 20]. Moreover, MCP-1 plasma levels have been extensively studied in gram-negative infections. High levels were found in experimental endotoxemia in baboons after sublethal and lethal doses of *Escherichia coli* [21]. As in this experimental study, elevated plasma levels of MCP-1 were found in humans with sepsis and septic shock; however, no differences were found between patients with gram-positive and gram-negative bacterial infections [22]. In contrast to these findings, we observed a trend for higher plasma MCP-1 levels in patients with gram-negative sepsis compared to the levels in those with gram-positive sepsis. Nevertheless, our observation is in line with findings in meningococcal sepsis or urosepsis—important gram-negative infections—that are associated with high MCP-1 concentrations in blood [23, 24].

Cortisol is an important sepsis biomarker. Elevated cortisol concentrations have been described in septic shock and gram-positive sepsis [25]. In contrast to this finding, we observed the highest cortisol levels in patients with gram-negative sepsis. This may be partly explained by a smaller proportion of BM patients (14%) in the gram-negative group compared to 37% of patients with BM in the gram-positive group, because the lowest cortisol levels in our study were found in BM patients. It is worth noting that the highest cortisol levels (>3000 nmol/mL) were observed in a patient with gram-negative sepsis arising from the biliary tract, in a patient with *E. coli* pneumonia complicated with venous thromboembolism (VTE), and in a patient with fulminant meningococcal sepsis who survived with severe sequelae (data not shown). Higher plasma cortisol levels have already been demonstrated in survivors of meningococcal sepsis in comparison to nonsurvivors, and in patients with Cushing's syndrome with increased risk of VTE; however, there have been no studies of cortisol levels in patients with biliary sepsis [26, 27]. It seems probable that these findings represent the severity of those clinical situations. Next, it has also been suggested that decreased plasma cortisol levels are not rare in septic shock patients and that poor outcomes cannot be predicted from cortisol levels in blood per se but rather by adrenocortical hyporesponsiveness [28]. Despite this paradigm, we observed a positive correlation between plasma cortisol levels and the daily SOFA score over the whole study period. Higher SOFA scores were found in septic patients with relative adrenal insufficiency when compared to patients with severe courses of sepsis without this alteration, but a direct association between the SOFA score and plasma cortisol levels was not reported [29]. It is well known that elevated cortisol levels may reflect the normal response of the hypothalamic-pituitary-adrenal (HPA) axis in sepsis, which is important for catecholamine effects and vasopressin release. Additionally, cortisol is responsible for maintaining the vascular response to catecholamines, leading to the maintenance of an adequate blood perfusion pressure of vital organs [30]. Interestingly, the elevation of cortisol levels in patients with IE is, to our knowledge, an original finding. Since the major difference was observed between patients with IE and those with BM, we can assume that the plasma cortisol levels were modulated by corticosteroid therapy, which is routinely administered to BM patients based on generally accepted guidelines and avoided in IE patients because of its relative contraindication [31]. In addition, we observed significantly higher cortisol levels in patients with IE than in those with CAP, which may also indicate an intense activation of the HPA axis during IE. Specific host reactions in IE are also supported by the observation of elevated plasma HBP levels at two time points in the study. It should be stressed that HBP may cause vascular injury and leakage in sepsis, and it may be possible that HBP plays the same role in bloodstream infections [32].

Increased serum levels of sCD14 have already been described in 54 patients with gram-negative septic shock with a median concentration of 3.23 mg/L, which was significantly higher than the median concentration in healthy controls (2.48 mg/L). In that study, using the ELISA method for sCD14 detection, the highest concentrations were observed in nonsurvivors in comparison to survivors—4.2 versus 2.8 mg/L, respectively [33]. Moreover, another study demonstrated that neonates with gram-negative sepsis had significantly higher sCD14 levels in their blood than did children with gram-positive sepsis [34]. We observed a similar trend in patients with gram-negative sepsis; however, it did not reach statistical significance. Additionally, we did not find a correlation between the severity of sepsis and the concentration of circulating sCD14. Similarly, no differences were found between the plasma sCD14 concentrations of survivors and nonsurvivors in a cohort of 142 adult patients with serious community-acquired infections [35]. Furthermore, not only the etiology but also the source of sepsis can influence the blood levels of sCD14. However, to our knowledge, there is a limited number of studies comparing plasma sCD14 levels in patients with different sources of sepsis arising from community-acquired infections. In our previous study aimed at identifying potential biomarkers of community-acquired bacterial and viral infections, no differences were found in serum sCD14 concentrations between patients with CAP and those with urosepsis [36]. The levels of sCD14 observed in the current study in patients with CAP seem to be higher than those detected in the previous study (4.50 versus 3.71 mg/L). This difference can reflect either the severity of disease, as the previous study was executed in the standard wards only, or the etiology of CAP, since some patients had mild-to-moderate pneumonia caused by atypical pathogens. Additionally, a blood type specimen could have played a role, because we compared levels in plasma and serum. Next, some studies reported similar sCD14 concentrations in patients with BM as those observed in the current study. For example, in one study, children with meningococcal meningitis and sepsis had a median plasma sCD14 concentration of 3.3 mg/L, which is only slightly higher than the levels found in our patients with BM [37]. The blood levels of sCD14 were also evaluated as a prognostic factor of CAP, and very high blood levels with a median concentration of 6.07 mg/mL were found in 198 survivors in comparison to the 6.70 mg/mL median level observed in nonsurvivors [38]. Since no information about the etiology of CAP was given in this study and a different scoring system was used for the assessment of the severity of pneumonia, it is impossible to compare these data with those of our study. We cannot compare our findings in patients with IE with those of other studies because, to our knowledge, there have been no studies performed about sCD14 levels in patients with IE. In addition, recent studies have frequently utilized a fragment of sCD14 denoted as sCD14-ST, also known as presepsin, suggesting that this glycoprotein is an interesting diagnostic sepsis biomarker [39].

Inflammatory cytokines have been extensively studied in sepsis. They have multiple functions during severe infections and sepsis, and some of them are considered reliable biomarkers that are useful for daily practice, especially in ICU patients. A good example is IL-6, the blood levels of which should correlate with the severity and bacterial etiology of sepsis [36, 40]. Based on these findings, IL-6 measurement has become routine in many clinical laboratories. However, in this study we observed an unacceptable IL-6 detectability, reaching approximately 82% on day 1 with a subsequent decrease to approximately 68% on days 2 and 3 to finally 60% on the last day of the study. A study with 70 septic shock patients demonstrated 64% detectability of IL-6 in sera at the study entry [41]. These data are relatively close to our finding on day 2 of the study, and they indicate the diagnostic limitation of IL-6 levels in septic patients. Another potential biomarker of sepsis is IL-8, which has strong chemoattractant and activating effects on neutrophils. Elevated plasma levels of this cytokine were found in septic patients, with a median concentration of 280 pg/mL, and IL-8 was detectable with the ELISA method in 89% of all examined specimens [42]. This detectability is very close to that found in our study, in which an average detectability of 86.2% was demonstrated over the study period. Sepsis is also associated with an intense anti-inflammatory response initiated almost simultaneously with the initial proinflammatory reactions. Therefore, IL-10, one of the strongest anti-inflammatory cytokines, can be detected in the blood during the acute phase of sepsis. In a study with 69 septic patients, the plasma levels of IL-10 were detectable with the ELISA method in heparinized blood in 39 (57%) of the enrolled subjects; the median IL-10 concentration was higher in patients with septic shock (58 pg/mL) in comparison to those with sepsis (11 pg/mL) [43]. If we compare our data with results of that study, we observed a lower detectability of IL-10, with a value of 46.6% compared to the 57% in the abovementioned study. Possible explanations of this difference could be that we did not examine specimens from patients with fatal outcomes or that we used the multiplex cytometric method for cytokine and chemokine detection. In any case, IL-10 is a less reliable biomarker than any other investigated inflammatory mediator in our study.

Our study has certain limitations. First, this is a single-center study from a highly specialized ICU for infectious disease patients, which is reflected in the relatively high proportions of specific infectious diseases—BM and IE. On the other hand, this also represents an advantage because of the high proportion of patients with established etiological diagnoses. Second, the patients were enrolled in the study the morning after their admission to the ICU; specimens collected immediately upon admission were only obtained from 30 subjects and therefore were not evaluated. Consequently, we did not assess the influence of antimicrobial therapy on the analyzed biomarkers. Third, we could not assess mortality and examine specimens from patients with fatal outcome, because the local ethics committee requested informed consent from relatives of succumbed patients, which we felt traumatic for their surviving family. Therefore, important data including mortality rate and differences in selected biomarkers among survivors and nonsurvivors are missing. Lastly, the groups of patients with evaluated infectious diseases and cohorts of patients with gram-positive and gram-negative infections were relatively small, and the primary aim of the study was not to test a diagnostic value of the selected biomarkers.

## 5. Conclusion

Altogether, our data indicate that MCP-1 and cortisol are promising biomarkers with a potential for differentiation between gram-positive and gram-negative sepsis and for evaluation of severity of the clinical course of sepsis. Moreover, it is apparent that blood levels of MCP-1, cortisol, sCD14, and HBP are significantly modulated by the source of sepsis, suggesting their pathophysiological and diagnostic importance, which should be further tested in a large cohort of septic patients.

## Figures and Tables

**Table 1:** Demographic and clinical data of septic patients.

	*n* = 62	
Age in years, median (interquartile range)	61 (70–46)	
Gender (male/female)	30/32	

Site of infection	*n*	(%)

Bacterial meningitis	18	29.0
Infective endocarditis	11	17.7
Community-acquired pneumonia	10	16.1
Urinary tract infection	6	9.7
Severe soft tissue infection	6	9.7
Biliary sepsis	4	6.5
Vertebral osteomyelitis	2	3.2
Meningococcal sepsis	1	1.6
Acute bacterial epiglottitis	1	1.6
Acute enteritis	1	1.6
Diverticulitis	1	1.6
Unknown origin	1	1.6

Etiology	*n*	%

Gram-positive bacteria	37	59.7
*Streptococcus pneumoniae*	16	25.8
Methicillin-sensitive *Staphylococcus aureus*	9	14.5
Methicillin-resistant *Staphylococcus aureus*	3	4.8
*Streptococcus agalactiae*	3	4.8
*Streptococcus pyogenes*	2	3.2
*Streptococcus viridans*	1	1.6
*Streptococcus mitis*	1	1.6
*Streptococcus anginosus*	1	1.6
*Listeria monocytogenes*	1	1.6
Gram-negative bacteria	14	22.6
*Escherichia coli*	5	8.1
*Klebsiella pneumoniae*	2	3.2
*Pseudomonas aeruginosa*	2	3.2
*Neisseria meningitidis*	2	3.2
*Haemophilus influenzae* non B	1	1.6
*Legionella pneumophila*	1	1.6
*Salmonella* group B	1	1.6
Polymicrobial infection	3	4.8
Methicillin-sensitive *Staphylococcus aureus*, *Peptostreptococcus* spp., *Bacteroides fragilis*	1	1.6
*Fusobacterium nucleatum*, *Porphyromonas* spp.	1	1.6
*Citrobacter freundi*, *Klebsiella oxytoca*	1	1.6
Virus	2	3.2
*Influenza A H1N1*	2	3.2
Pathogen not identified	6	9.7

**Table 2:** The comparison of baseline characteristics of patients with gram-positive (*n* = 37) and gram-negative (*n* = 14) sepsis.

	Gram positive	Gram negative	*P*
	*n* = 37	*n* = 14	
Age, years	59 (66–42)	69.5 (75–35)	0.485
Gender (male/female)	19/18	6/8	—
APACHE II	16 (21–13)	21 (24–14)	0.261
SOFA	6 (5–8)	7.5 (6–9)	0.182
WBC (cells/*μ*L)	16,400 (24,600–10,250)	14,750 (29,000–9100)	0.883
CRP (mg/L)	263 (319–179)	181 (267–111)	**0.026**

Data are expressed as medians (interquartile range). APACHE II: Acute Physiology and Chronic Health Evaluation II; SOFA: Sequential Organ Failure Assessment; WBC: white blood cell count; CRP: C-reactive protein.

**Table 3:** The comparison of baseline characteristics of patients with bacterial meningitis (*n* = 18), infective endocarditis (*n* = 11), and community-acquired pneumonia (*n* = 10).

	Bacterial meningitis	Infective endocarditis	Community-acquired pneumonia	*P*
	*n* = 18	*n* = 11	*n* = 10	
Age, years	64 (69-70)	59.5 (32–66)	48 (33–62)	0.140
Gender (male/female)	7/11	5/6	6/4	
APACHE II	18.5 (16–21)	10.5 (8–17)	21.5 (23.25–13.75)	0.038
SOFA	6.5 (5–8)	5.5 (2–7.5)	7 (5–8)	0.109
WBC (cells/*μ*L)	22,000 (15,000–29,500)	11,250 (9400–20,550)	9600 (15,825–6050)	0.410
CRP (mg/L)	187 (119–312)	282 (243–313)	288 (414–107.5)	0.972

Data are expressed as medians (interquartile range). APACHE II: Acute Physiology and Chronic Health Evaluation II; SOFA: Sequential Organ Failure Assessment; WBC: white blood cell count; CRP: C-reactive protein.

**Table 4:** The etiology of bacterial meningitis (*n* = 18), infective endocarditis (*n* = 11), and community-acquired pneumonia (*n* = 10).

	*n* (%)
Bacterial meningitis	18
*Streptococcus pneumoniae*	11 (61)
*Streptococcus agalactiae*	1 (5.6)
*Listeria monocytogenes*	1 (5.6)
*Neisseria meningitidis*	1 (5.6)
*Fusobacterium nucleatum*, *Porphyromonas* spp.	1 (5.6)
*Haemophilus influenzae* non B	1 (5.6)
Pathogen not identified	2 (11)
Infective endocarditis	11
*Staphylococcus aureus* (methicillin sensitive)	7 (63.6)
*Staphylococcus aureus* (methicillin resistant)	1 (9.1)
*Streptococcus pyogenes*	1 (9.1)
*Streptococcus mitis*	1 (9.1)
*Streptococcus viridans*	1 (9.1)
Community-acquired pneumonia	10
*Streptococcus pneumoniae*	5 (50)
*Legionella pneumoniae*	1 (10)
*Escherichia coli*	1 (10)
Influenza A	2 (20)
Pathogen not identified	1 (10)

**Table 5:** Total and system-related SOFA score values in the groups of patients evaluated based on source and etiology of sepsis.

System	SOFA score				
	Bacterial meningitis *n* = 18	Infective endocarditis *n* = 11	Community-acquired pneumonia *n* = 10	Gram-positive sepsis *n* = 37	Gram-negative sepsis *n* = 14
Respiration	1.5 (0–2)	0 (0–2)	3 (2-3)	1 (0–2)	2 (0–2)
Coagulation	0 (0-1)	1 (0–3)	0 (0-1)	1 (0-1)	0 (0–2)
Liver	0 (0–0)	0 (0-1)	0 (0-1)	0 (0-1)	0 (0–2)
Cardiovascular	0.5 (0–2)	0 (0–2)	2 (1-2)	0 (0–2)	2 (0–2)
Central nervous system	3 (3–3)	0 (0–2)	0 (0-1)	1 (0–3)	0 (0–2)
Renal	0 (0-1)	1 (0-1)	2 (0–4)	0 (0-1)	1.5 (0–2)
SOFA total	6.5 (5–8)	5.5 (2–7.5)	7 (5–8)	6 (5–8)	7.5 (6–9)

Data are presented as medians (interquartile range). SOFA: Sequential Organ Failure Assessment.

**Table 6:** Detectability of laboratory markers during the study.

	Day 1		Day 2		Day 3		Day 4		Total (4 days)	
Parameter	Detected/total		Detected/total		Detected/total		Detected/total		Detected/total	
MIP-1*β*	57/61	93.4%	58/62	93.5%	56/62	90.3%	58/62	93.5%	229/247	92.7%
MCP-1	61/61	100%	62/62	100%	62/62	100%	62/62	100%	247/247	100%
IL-10	36/61	59.0%	28/62	45.2%	27/62	43.5%	24/62	38.7%	115/247	46.6%
IL-8	54/61	88.5%	53/62	85.5%	52/62	83.9%	54/62	87.1%	213/247	86.2%
IL-6	50/61	82.0%	46/62	67.7%	42/62	67.7%	37/62	59.7%	175/247	70.9%
HBP	57/57	100%	59/59	100%	59/59	100%	59/59	100%	234/234	100%
sCD14	61/61	100%	62/62	100%	62/62	100%	62/62	100%	247/247	100%
Cortisol	56/56	100%	57/58	98.3%	57/59	96.6%	57/59	96.6%	227/232	97.8%

**Table 7:** The comparison of biomarker levels between gram-positive and gram-negative sepsis.

Parameter	Day 1		Day 2		Day 3		Day 4		*P*
	G+	G−	G+	G−	G+	G−	G+	G−	
MCP-1 (pg/mL)	576.40 (1188.61)	685.84 (4291.12)	505.33 (692.46)	786.23 (1593.18)	450.84 (742.37)	715.29 (848.93)	415.96 (438.42)	745.97 (616.07)	n.s.
MIP-1*β* (pg/mL)	43.49 (62.26)	48.99 (75.74)	43.03 (79.45)	39.39 (67.09)	39.11 (72.34)	28.45 (65.29)	37.78 (86.18)	36.79 (47.61)	n.s.
IL-6 (pg/mL)	47.74 (133.21)	18.96 (399.91)	20.98 (56.49)	27.98 (202.13)	11.28 (40.93)	11.21 (295.56)	2.65 (40.24)	15.02 (41.50)	n.s.
IL-8 (pg/mL)	80.54 (192.72)	106.62 (150.94)	60.14 (187.78)	31.43 (89.09)	65.83 (174.18)	40.56 (107.72)	63.23 (208.04)	34.49 (98.74)	n.s.
IL-10 (pg/mL)	6.32 (15.18)	1.90 (47.42)	1.90 (8.84)	3.64 (6.48)	1.90 (12.34)	1.90 (15.91)	1.90 (8.44)	1.90 (3.94)	n.s.
HBP (ng/mL)	236.33 (462.47)	288.59 (894.53)	122.42 (267.37)	162.18 (532.89)	111.85 (238.57)	124.47 (232.94)	82.94 (113.00)	115.96 (388.1)	n.s.
sCD14 (mg/L)	3.62 (1.34)	3.89 (1.12)	3.59 (1.48)	4.11 (1.14)	3.58 (1.39)	3.82 (1.24)	3.68 (2.24)	3.28 (1.64)	n.s.
Cortisol (nmol/L)	326.90 (290.20)	442.80 (400.25)	396.20^∗^ (421.4)	662.00^∗^ (2038.60)	423.20 (431.20)	545.55 (538.2)	452.60 (335.5)	416.15 (381.50)	**0.048**

Data are presented as median (quartile); n.s.: not significant; ^∗^*P* < 0.05: gram-positive (G+) versus gram-negative (G−) infection. The statistical difference-analyzed parameters in the groups were analyzed by the Mann–Whitney *U* test.

**Table 8:** The comparison of biomarker levels among the groups based on the source of sepsis.

Parameter	Day 1			Day 2			Day 3			Day 4		
	BM	IE	CAP	BM	IE	CAP	BM	IE	CAP	BM	IE	CAP
MCP-1 (pg/mL)	133.8^1,2^ (149.0)	542.4^1^ (693.2)	685.6^2^ (1064.9)	130.0^1,2^ (158.5)	624.5^1^ (454.3)	667.7^2^ (509.3)	154.1^1,2^ (136.6)	489.5^1^ (274.4)	581.5^2^ (513.5)	221.1^1,2^ (216.9)	471.8^1^ (334.4)	605.3^2^ (378.9)
MIP-1*β* (pg/mL)	25.0 (67.0)	40.6 (56.7)	23.1 (73.4)	14.2 (63.7)	29.7 (74.8)	12.8 (36.2)	16.4 (45.8)	30.1 (82.4)	13.8 (22.5)	23.3 (73.9)	26.9 (90.9)	13.4 (26.4)
IL-6 (pg/mL)	12.8 (46.5)	22.1 (113.8)	49.9 (191.9)	1.6^1,2^ (10.4)	29.9^1^ (76.3)	18.4^2^ (91.2)	1.2^1,2^ (6.9)	29.9^1^ (61.0)	8.1^2^ (96.0)	1.2 (12.1)	42.5 (92.1)	1.2 (25.9)
IL-8 (pg/mL)	13.1 (133.7)	37.3 (157.4)	47.1 (102.9)	14.2 (169.6)	34.7 (126.8)	22.6 (43.5)	32.3 (151.1)	33.6 (191.1)	34.4 (99.5)	44.7 (130.9)	52.4 (185.5)	25.1 (34.0)
IL-10 (pg/mL)	1.9^1,2^ (0.0)	11.9^1^ (17.7)	10.2^2^ (6.6)	1.9^1^ (0.0)	9.8^1^ (14.4)	1.9 (7.6)	1.9^1^ (0.0)	5.2^1^ (14.5)	9.5 (13.3)	1.9^2^ (0.0)	1.9 (7.2)	12.5^2^ (14.2)
HBP (ng/mL)	110.1^1^ (225.1)	369.6^1^ (826.3)	96.7 (187.0)	92.1 (165.4)	189.5 (362.6)	57.2 (140.4)	66.8 (134.5)	230.2 (457.0)	102.5 (130.8)	43.4 (77.1)	111.3 (224.8)	155.2 (332.8)
sCD14 (mg/L)	3.0^2^ (1.0)	4.0 (1.3)	4.5^2^ (0.6)	2.8^1,2^ (0.6)	4.2^1^ (1.2)	4.0^2^ (1.1)	2.6^1,2^ (0.5)	3.9^1^ (0.8)	3.7^2^ (2.1)	2.3^1,2^ (1.5)	4.0^1^ (1.6)	4.2^2^ (2.9)
Cortisol (nmol/L)	174.3 (146.9)	556.2 (531.9)	324.7 (555.8)	95.1^1^ (75.7)	509.5^1^ (450.8)	290.6 (377.4)	76.7^1,2^ (88.7)	528.7^1^ (217.8)	215.1^2^ (329.3)	111.3^1^ (248.5)	531.5^1,3^ (143.3)	220.6^3^ (305.6)

Data are presented as median (quartile); BM: bacterial meningitis; IE: infective endocarditis; CAP: community-acquired pneumonia; ^1^*P* < 0.05: BM versus IE; ^2^*P* < 0.05: BM versus CAP; ^3^*P* < 0.05: IE versus CAP. The statistical difference-analyzed parameters for multiple comparison were analyzed by the Kruskall-Wallis test.

**Table 9:** The relationship of biomarker levels and SOFA score during the study.

Parameter	SOFA Day 1	SOFA Day 2	SOFA Day 3	SOFA Day 4
MCP-1 (pg/mL)	0.425^∗^	0.510^∗^	0.355^∗^	0.440^∗^
Cortisol (nmol/L)	0.336^∗^	0.382^∗^	0.499^∗^	0.415^∗^
sCD14 (mg/L)	0.061	0.074	0.118	0.237^∗^
MIP-1*β* (pg/mL)	0.039	0.107	0.081	0.085
HBP (ng/mL)	−0.032	−0.021	−0.108	−0.140

^∗^ *P* < 0.05. Spearman's correlation test was used for determination of correlations. SOFA: Sequential Organ Failure Assessment.

## Data Availability

Requests for data, 12 months after initial publication, will be considered by the corresponding author.
